# Data on cytotoxic pattern of cholesterol analogs for lung adenocarcinoma cells

**DOI:** 10.1016/j.dib.2019.104179

**Published:** 2019-06-25

**Authors:** Yamixa Delgado, Anamaris Torres, Melissa Milian

**Affiliations:** Laboratory of Drug Design & Delivery, Biochemistry and Pharmacology Department, San Juan Bautista School of Medicine, USA

## Abstract

Cholesterol (Cho) is a sterol that plays an essential role in the maintenance of biologic cell membranes, and various lipoproteins are its carriers through blood circulation [1]. Some FDA-approved anticancer drugs (i.e., Lipoplatin and Myocet) are conjugated to Cho moieties to improve their pharmacokinetic properties, cellular uptake and target specificity [2]. Recently natural and synthetic sterol compounds have shown a broad spectrum of pharmacological activities [3,4]. Herein, we investigated the anticancer activity of various natural Cho analogs, ie. asiatic acid (AsA), betulinic acid (BeA), oleanolic acid (OleA), ursolic Acid (UrA), lupeol (Lupe) and β-sitosterol (β-Sito) against non-small cell lung adenocarcinoma (A549). We performed theoretical calculations of the biophysicochemical properties, and viability assays in a range of 5–100 μM in A549 cells of these Cho analogs. We used ChemSketch and ChemSpider to determine physical properties, and GraphPad Prism 8 software for the data analysis to determine the inhibitory concentrations at 50% (IC_50_) of each compound.

**Table:** Specifications Table

Subject area	*Biochemistry*
More specific subject area	*Biological role of plant-derived sterols*
Type of data	*Graphs, images and figures*
How data was acquired	*Physicochemical properties calculations: ChemSketch and ChemSpider MTS viability assay: CellTiter 96® AQueous MTS Reagent Powder (Promega), Phenazine methosulfate (PMS) (Millipore Sigma); Light microscope (Nikon TMS), microplate reader (Fisher Scientific Multiscan FC)).*
Data format	*Analyzed*
Experimental factors	*10 mMstocks solutions of the six cholesterol (Cho) analogs, of cisplatin (positive control) and of Cho (negative control) were prepared in DMF solvent the same day of the cellular treatment. The compounds were incubated at different concentrations (5, 10, 25, 50, 75 and* 100 μM*) in A549 cells at 37°C and 5% CO*_*2*_*for 24h.*
Experimental features	*Cytotoxicity was determined by adding MTS/PMS reagent and measuring the colorimetric intensity after 24 h treatment with Cho analogs and controls.* *Graphics and IC*_*50*_*values determination were performed using GraphPad Prism 8 software.*
Data source location	*Caguas, Puerto Rico, USA*
Data accessibility	*All data are presented in this article.*
Related research article	*C.M. Wang, K.L. Yeh, S.H. Tsai* et al. *Anti-proliferative activity of triterpenoids and sterols isolated from Alstonia scholaris against non-small-cell lung carcinoma cancer, Molecules 22 (2017) 2119.*

**Table:** 

**Value of the data** • The data show the bioactivity of plant-derived Cho analogs in A549 cancer cells. • The data are useful for synthetic chemists working on the development of anticancer drugs because they can create connections and comparisons between the potential drug and the proposed Cho-like structure analogs based on the calculated physicochemical properties. • The data indicate that five of the Cho analogs (UrA, BeA, OleA, AsA, and Lupe) showed cytotoxic effect at the micromolar range tested. However BeA showed the more potent cytotoxic pattern with the lowest IC50. On the contrary, β–sito did not show any significant cytotoxicity. • In the methodology, a protocol is provided to easily screen different compounds for their cytotoxic patterns by MTS viability assay using CellTiter 96® AQueous MTS Reagent Powder and Phenazine methosulfate (PMS). • A protocol to create graphics, analyze data and determine IC_50_ values using GraphPad Prism 8 software.

## 1 Data

**Fig. 1:**
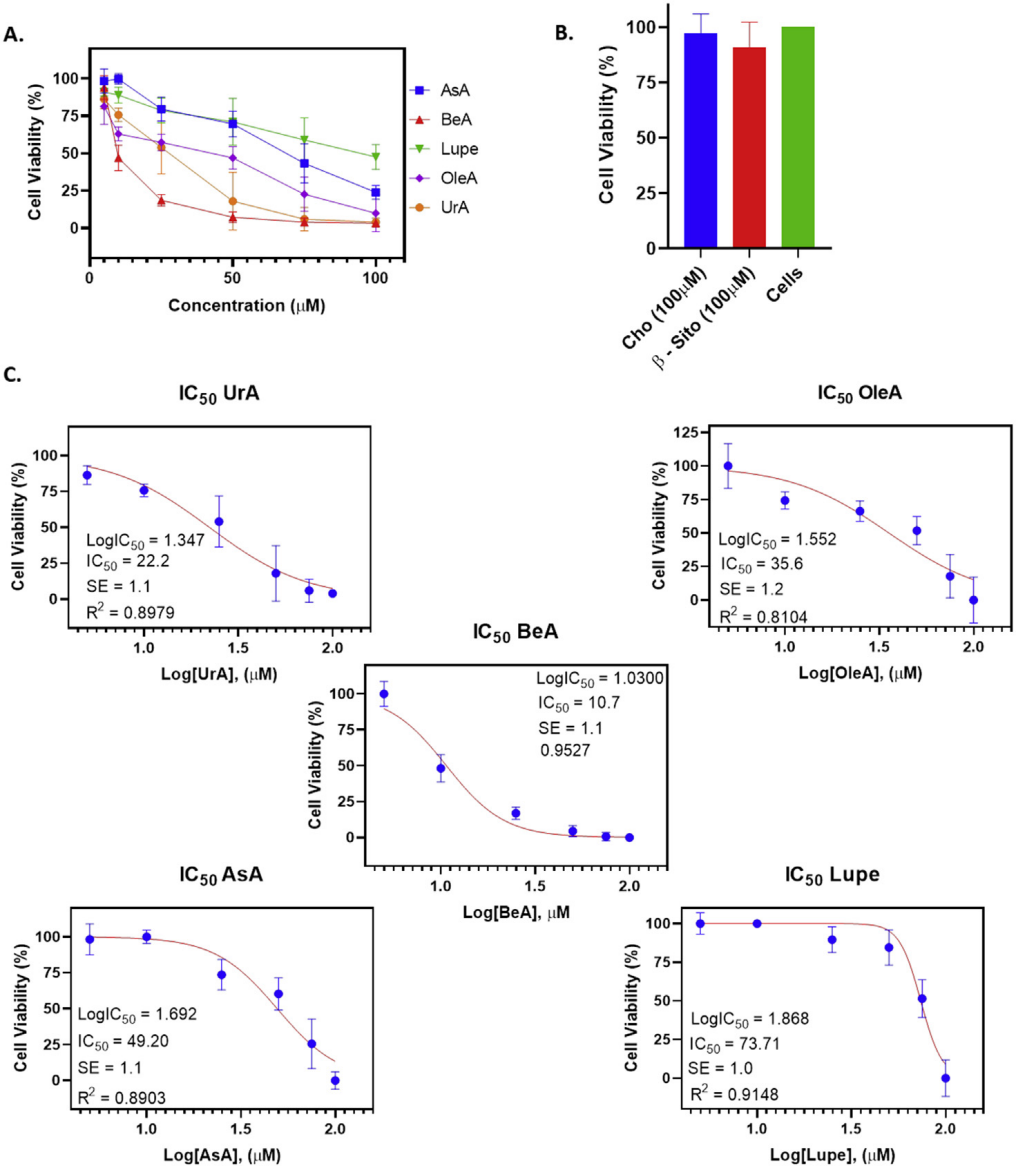
Determination of IC50 values of the Cho analogs for 24 h of incubation in A549 cells using GraphPad Prism 8. **A.** Results of the viability assays of the Cho analogs; **B.** Results of the controls: 100 μM Cho and β-Sito. **C.** Analysis generated by the software for the determination of the IC_50_ values.

In this report, we present data on the cytotoxicity of Cho analogs: UrA, BeA, OleA, AsA, Lupe and β-Sito (structures in Table 1). Our Cho analogs are commercial plant-derived triterpenoids with Cho fundamental structure. Some of them (e.g., BeA, OleA and UrA) have shown anti-tumorigenic and antibacterial properties [3], [4], [5], [6]. In the Table 1 we theoretically determined some of the physicochemical properties of the six Cho analogs compared to Cho, following some of the most important factors to overcome physiological barriers. The six analogs show bioavailability potential due to the high lipophilicity (LogP) in the same way as the Cho but with more ionizable groups (LogD). It is suggested that high lipophilic drugs will accumulate to a high concentration within the cellular membrane changing its fluidity and promoting cell death [1], [2], [7]. The cytotoxic effects of these Cho analogs were determined at different concentrations (5, 10, 25, 50, 75 and 100 μM) in the A549 cells after 24 h treatment. Viability of cells was measured by the colorimetric absorbance at 492 nm of the formazan dye produced after the addition of the MTS/PMS reagent. The intensity of the produced dye is correlated to the reduction of the MTS molecule, assisted by electron coupler PMS, by the NADH-dependent cellular oxidoreductase enzymes to generate a colored formazan product. The raw data and normalized values (after subtracting the background) are shown in the Table 2. Graphics of the normalized data and IC_50_ values determinations of the compounds are shown in the Fig. 1. UrA, BeA, OleA, AsA and Lupe showed cytotoxic patterns in the micromolar range tested in this study. However, β-Sito did not show any significant cytotoxicity even at the highest concentration of 100μM.

**Table 1:** Physicochemical properties calculations of the Cho analogs.

Cho Analogs*Empirical Formula*	Physicochemical Properties							
	Structure^a^	MW^b^ (Da)	H-Bond Acceptor^b^	H-Bond Donor^b^	Aqueous Solubility^b^ (mg/L) 25 °C	LogP^a^	LogD^b^	A^b^ (cm^3^)
**AsA** *C*_*30*_*H*_*48*_*O*_*5*_		488.7	5	4	6.0 × 10^−2^	6.5	3.5	137
**UrA** *C*_*30*_*H*_*48*_*O*_*3*_		456.7	3	2	1.9 × 10^−3^	9.0	5.9	134
**BeA** *C*_*30*_*H*_*48*_*O*_*3*_		456.7	3	2	1.6 × 10^−3^	8.9	5.1	133
**OleA** *C*_*30*_*H*_*48*_*O*_*3*_		456.7	3	2	1.7 × 10^−3^	9.0	5.9	134
**Lupe** *C*_*30*_*H*_*50*_*O*		426.7	1	1	8.8 × 10^−5^	11.0	9.4	132
**β-Sito** *C*_*29*_*H*_*50*_*O*		414.7	1	1	4.6 × 10^−5^	10.7	9.4	129
**Cho** *C*_*27*_*H*_*46*_*O*		386.6	1	1	4.1 × 10^−4^	9.9	8.1	120

MW: molecular weight; LogP: Lipophilicity; LogD: Lipophilicity considering ionizable groups; A: Molar Refractivity.

a Generated using ChemSketch by ACD/Labs.

b Generated using ChemSpider.

**Table 2:** Values shown in the table are percentiles of cell viability averages according to the concentrations of three different independent experiments. Background control was used to normalize the raw data. IC_50_ are also included in the table. CisPt and Cho were used as positive and negative controls respectively. Cell viability averages (Mean) and standard deviations (SD) are shown in bold. The inhibitory concentration at 50%(IC50) and the standard error (SE) are shown in bold and italic.

Cho analogs	A549 cells	Concentration (μM)					
		5	10	25	50	75	100
**UrA**	Exp.1	93 ± 16	80 ± 13	74 ± 10	40 ± 15	15 ± 6	3 ± 0
	Exp. 2	80 ± 12	76 ± 4	48 ± 8	10 ± 8	3 ± 1	7 ± 1
	Exp. 3	86 ± 4	71 ± 7	40 ± 4	4 ± 6	0 ± 0	2 ± 0
	**Mean ± SD** ***IC***_***50***_***± SE = 28±1 μM***	**86 ± 7**	**76 ± 5**	**54 ± 18**	**18 ± 18**	**6 ± 8**	**4 ± 3**
**BeA**	Exp.1	97 ± 8	56 ± 7	17 ± 3	7 ± 2	5 ± 1	3 ± 1
	Exp. 2	85 ± 5	46 ± 8	23 ± 0	11 ± 3	6 ± 2	5 ± 1
	Exp. 3	100 ± 5	39 ± 8	16 ± 0	4 ± 3	1 ± 1	2 ± 1
	**Mean** ± **SD** ***IC***_***50***_***± SE = 11****±1* ***μM***	**94 ± 6**	**47 ± 7**	**19 ± 4**	**7 ± 3**	**4 ± 3**	**3 ± 2**
**OleA**	Exp.1	73 ± 5	68 ± 1	60 ± 16	46 ± 10	23 ± 4	24 ± 4
	Exp. 2	76 ± 8	59 ± 11	51 ± 3	55 ± 2	11 ± 5	1 ± 1
	Exp. 3	95 ± 3	62 ± 2	61 ± 2	40 ± 5	34 ± 3	5 ± 3
	**Mean** ± **SD** ***IC***_***50***_***± SE = 36±1 μM***	**81 ± 9**	**63 ± 5**	**57 ± 6**	**47 ± 8**	**23 ± 12**	**10 ± 12**
**AsA**	Exp.1	97 ± 5	100 ± 4	87 ± 10	73 ± 5	42 ± 9	20 ± 4
	Exp. 2	91 ± 9	96 ± 3	88 ± 15	76 ± 11	57 ± 11	23 ± 3
	Exp. 3	107 ± 2	103 ± 5	71 ± 6	60 ± 3	31 ± 9	29 ± 5
	**Mean** ± **SD** ***IC***_***50***_***± SE = 49±1*** ***μM***	**98 ± 8**	**100 ± 4**	**79 ± 15**	**66 ± 14**	**43 ± 13**	**24 ± 4**
**Lupe**	Exp.1	104 ± 6	99 ± 6	94 ± 6	87 ± 2	70 ± 3	21 ± 3
	Exp. 2	97 ± 5	96 ± 5	93 ± 8	94 ± 6	51 ± 10	26 ± 1
	Exp. 3	93 ± 2	99 ± 4	82 ± 3	76 ± 3	57 ± 7	8 ± 2
	**Mean** ± **SD** ***IC***_***50***_***± SE = 72±1*** ***μM***	**98 ± 6**	**98 ± 2**	**90 ± 7**	**86 ± 9**	**59 ± 9**	**18 ± 9**
**β-Sito**	Exp. 1	–	–	–	89 ± 5	–	80 ± 2
	Exp. 2	–	–	–	97 ± 4	–	90 ± 11
	Exp. 3	–	–	–	100 ± 1	–	88 ± 5
	**Mean** ± **SD**	–	–	–	**95 ± 6**	–	**86 ± 5**
**CisPt (** + **Control)**	Exp. 1	–	–	–	43 ± 2	–	–
	Exp. 2	–	–	–	63 ± 4	–	–
	Exp. 3	–	–	–	49 ± 1	–	–
	**Mean** ± **SD**	–	–	–	**52 ± 9**	–	–
**Cho (- Control)**	Exp. 1	–	–	–	98 ± 5	–	87 ± 3
	Exp. 2	–	–	–	94 ± 6	–	94 ± 3
	Exp. 3	–	–	–	101 ± 4	–	100 ± 1
	**Mean** ± **SD**	–	–	–	**98 ± 4**	–	**94 ± 7**
**Background control**	Exp. 1	0 ± 1	1 ± 1	0 ± 0	2 ± 1	1 ± 0	0 ± 0
	Exp. 2	1 ± 0	1 ± 1	0 ± 1	1 ± 1	1 ± 1	1 ± 2
	Exp. 3	0 ± 0	0 ± 1	1 ± 0	1 ± 0	2 ± 0	0 ± 1
	**Mean** ± **SD**	**0 ± 1**	**1 ± 1**	**0 ± 1**	**1 ± 1**	**1 ± 1**	**0 ± 1**

## 2 Experimental design, materials, and methods

### 2.1 Materials

Aqueous solutions were prepared with sterile (autoclave conditions: 121 °C and 18 PSI) high quality nanopure water (18.2 MΩ cm resistivity, Thermoscientific^®^ Easypure water purifier). The non-small lung human adenocarcinoma A549 cell line (ATCC^®^ CCL-185™) was purchased from American Type Culture Collection (ATCC; Manassas, VA). Dulbecco's modified eagle medium (DMEM), phosphate buffer saline (PBS), fetal bovine serum (FBS), phenazine methosulfate (PMS), penicillin/streptomycin antibiotic solution and Cho analogs (UrA, BeA, OleA, AsA, Lupe and β-sito) and Cho were ordered from Millipore Sigma (St. Louis, MO). *CellTiter 96® AQueous MTS Reagent Powder* was ordered from Promega. All other chemicals were of analytical grade and from various commercial suppliers and used without further purification.

### 2.2 Cell culture

A549 cells were maintained in accordance with the ATCC protocol. Briefly, cells were cultured in 75 cm^2^ flasks with DMEM supplemented with 10% heat inactivated FBS and 1% antibiotic solution in a humidified incubator at 5% CO_2_ and 37 °C. All experiments were conducted before cells reached 30 passages. In each passage, cells were washed twice with PBS, trypsinized, and suspended in supplemented medium.

### 2.3 Viability assay

The cell viability of the A549 line, after being treated with the Cho analogs, was determined using the CellTiter 96^®^ Aqueous MTS Reagent Powder (Promega). A549 cells were seeded in a 96-well plate at a density of 5 × 10^3^ cell/well and then incubated for 24 hours at 37 °C and 5% CO_2_. Stocks of the Cho analogs: AsA, BeA, UrA, OleA, Lupe and β-Sito, were prepared at 10 mM in 1 mL of DMF. Dilutions of the Cho analogs were prepared in PBS to treat the cells at a range of 5, 10, 25, 50, 75 and 100 μM maintaining 1% of DMF in each well completing to a total volume of 200 μL. In non-treated cells, we added PBS and 1% DMF as a negative control and for background control, PBS and 1% DMF in wells without cells, to complete to the same total volume of 200 μL. Afterwards, the treated plate was incubated with the different compounds for 24 h. Following the incubation period and previous to the addition of the MTS/PMS solution, the plate was measured to obtain the background absorbances at 492 nm using a microplate reader spectrophotometer (Thermoscientific Multiskan FC). Then, 20 μL of MTS/PMS sterile solution [2 mg/mL MTS/0.21 mg/mL PMS] were added to each well followed by 1 hour of incubation in the dark at 37 °C. Then, the absorbance at 492 nm was measured. Background absorbances were subtracted of the sample absorbances after the incubation with the MTS/PMS. Cisplatin (CisPt) at 50 μM was used as the positive control and Cho as the negative control. The relative cell viability (%) was calculated by:

$$Relative\;cell\;viability\;\left(\%\right)=\frac{Abs\;treated\;cells}{Abs\;nontreated\;cells}x\;100$$

Cho analogs-treated cells at their respective IC_50_ were visualized under the microscope to confirm the results of the MTS/PMS assay.

### 2.4 Statistical analysis

Viability results are reported as average ± SD of at least three independent experiments. IC_50_ values and graphics were done using GraphPad Prism 8 software using the inhibitor vs normalize response method.
